# Screening of Antifungal and Antibacterial Activity of 90 Commercial Essential Oils against 10 Pathogens of Agronomical Importance

**DOI:** 10.3390/foods9101418

**Published:** 2020-10-07

**Authors:** Caroline De Clerck, Simon Dal Maso, Olivier Parisi, Frédéric Dresen, Abdesselam Zhiri, M. Haissam Jijakli

**Affiliations:** 1 Integrated and Urban Plant Pathology Laboratory, Gembloux Agro-Bio Tech (Liege University), Passage des Déportés 2, 5030 Gembloux, Belgium; S.DalMaso@uliege.be (S.D.M.); oparisi@gmail.com (O.P.); Frederic.Dresen@uliege.be (F.D.); 2 Pranarom International, Avenue des Artisans 37, 7822 Ghislenghien, Belgium; azhiri@pranarom.com

**Keywords:** essential oil, biocontrol, antifungal, antibacterial, biopesticide

## Abstract

Nowadays, the demand for a reduction of chemical pesticides use is growing. In parallel, the development of alternative methods to protect crops from pathogens and pests is also increasing. Essential oil (EO) properties against plant pathogens are well known, and they are recognized as having an interesting potential as alternative plant protection products. In this study, 90 commercially available essential oils have been screened in vitro for antifungal and antibacterial activity against 10 plant pathogens of agronomical importance. EOs have been tested at 500 and 1000 ppm, and measures have been made at three time points for fungi (24, 72 and 120 h of contact) and every two hours for 12 h for bacteria, using Elisa microplates. Among the EOs tested, the ones from *Allium sativum*, *Corydothymus capitatus*, *Cinnamomum cassia*, *Cinnamomum zeylanicum*, *Cymbopogon citratus*, *Cymbopogon flexuosus*, *Eugenia caryophyllus*, and *Litsea citrata* were particularly efficient and showed activity on a large panel of pathogens. Among the pathogens tested, *Botrytis cinerea*, *Fusarium culmorum*, and *Fusarium graminearum* were the most sensitive, while *Colletotrichum lindemuthianum* and *Phytophthora infestans* were the less sensitive. Some EOs, such as the ones from *A. sativum*, *C. capitatus*, *C. cassia*, *C. zeylanicum*, *C. citratus*, *C. flexuosus*, *E. caryophyllus*, and *L. citrata*, have a generalist effect, and are active on several pathogens (7 to 10). These oils are rich in phenols, phenylpropanoids, organosulfur compounds, and/or aldehydes. Others, such as EOs from *Citrus sinensis*, *Melaleuca* *cajputii*, and *Vanilla fragrans*, seem more specific, and are only active on one to three pathogens. These oils are rich in terpenes and aldehydes.

## 1. Introduction

Fruits, vegetables, and cereals are important components of the human diet at every age [1]. The increased demand for these commodities exert significant pressure on the environment, leading to intensive agriculture and the use of chemical pesticides. However, the use of these chemicals, and the resulting presence of their residues in food and water, are leading to several health safety breakdowns. Moreover, the use of chemical pesticides affects the environment and the biodiversity. The constant (and sometimes inadequate) use of pesticides is also responsible of the development of pathogen resistances leading to possible food safety issues [2].

Today, the demand for a reduction of chemical pesticides, and for the development of alternative ways to protect crops from pathogens and pests, is growing [3]. In response, research and development in the field of biopesticides has grown exponentially in the last 20 years.

Among the natural alternatives to chemical pesticides, products based on plant extracts and/or plant essential oils (EOs) have received increasing attention because of their generally recognized as safe (GRAS) compounds, due to their very low human toxicity, high volatility, and rapid degradation [4].

Essential oils possess a strong odor and are produced by aromatic plants as secondary metabolites [5]. They are usually obtained from several plant parts by steam hydrodistillation [6]. They are made of a mixture of volatile compounds (between 20 and 100), even if they are, in most cases, characterized by two or three main compounds, representing the major part of the EO (20–70%). As an example, EO of *Citrus limon* is composed, in majority, of limonene and β-pinene [7,8]. Two kinds of molecules can enter in the composition of essential oils: terpenes and terpenoids (e.g., limonene, linalool); and aromatic and aliphatic molecules (e.g., cinnamaldehyde, safrole) [9]. All of these components are characterized by a low molecular weight [10].

Essential oils were known, for a long time, for their antimicrobial and medicinal properties. The latter have, among others, led to the development of aromatherapy, where they are used as bactericide (e.g., tea tree and cinnamon EOs), fungicide (*Lavandula spica* EO), or virucid (*Cinnamomum camphora*) [5,11].

In the last 20 years, the antibacterial and antifungal properties of essential oils have been assessed against a large variety of plant pathogens in order to determine their potential as alternative plant protection products [6,12]. The complex composition of essential oils is interesting, as they could act as multisite chemicals, lowering the risk of resistance [13].

Furthermore, essential oils are composed by low molecular weight molecules and are highly volatile. This property is of great interest, particularly when used on fresh products or during postharvest applications. However, this advantage, in terms of residue reduction, is also a major inconvenience for crop application, which has to be overcome by a formulation allowing to maintain the efficacy of the product [14].

In this study, the in vitro efficacy of 90 commercially available essential oils against 10 plant pathogens of agronomical importance has been assessed. This is, to our knowledge, the largest screening for antifungal and antibacterial activity of EOs made so far.

## 2. Materials and Methods 

### 2.1. Essential Oils

The 90 essential oils (EOs) tested in our study were supplied by Pranarom International (Ghislenghien, Belgium) (Table 1).

### 2.2. Fungal and Bacterial Strains

The 10 host–pathogen combinations used in this study are listed in Table 2. All of the cultures were carried out at a 16D:8N photoperiod on the most appropriate solid media (see Table 2). The Potato dextrose agar (PDA) (Merck) medium was prepared according to the manufacturer’s instructions (39 g of powder in 1 L of water). The Luria-Bertani-agar (LB-agar) medium was composed of 10 g/L of peptone 5g/L of yeast extract, 10g/L of NaCl, and 15 g/L of agar. The V8 medium was made with 100 mL/L of V8 juice, 200 mg/L of CaCO_3_, and 20 g of agar. For in vitro screening procedures in liquid medium, pathogens have been cultured in the same media without the addition of agar. All of the media were autoclaved during 20 min at 120 °C.

### 2.3. Making of a Stable EO Emulsion

EOs are not water soluble. In order to get homogenous and stable emulsions, a formulation was developed to get a final EO concentration of 1000 ppm (maximum dose tested in the in vitro screening). The EOs were first diluted in ethanol in a ratio of 16.7:83.3%. Half a milliliter of this solution was then mixed with 555 µL of Tween 20 and 26.71 mL of distilled water, in order to get an EO concentration of 0.3%. For the in vitro screening procedure, this emulsion was diluted to reach the desired final EO concentration (see Section 2.4.2).

### 2.4. In Vitro Screening Procedure

#### 2.4.1. Determination of the Pathogens Kinetic Growth

The aim of this step was to determine the optimal growth conditions for each of the pathogens tested (exponential growth phase between 0 h and 48 h, followed by a growth plateau).

The kinetic growth of each pathogen in liquid media was determined using 96 wells ELISA microplates, following the method developed and validated by [15]. Three dilutions (3x, 30x, and 300x) of the medium and three concentrations (10^4^, 10^5^, and 10^6^ spores/mL) of spores’ suspensions were tested for each fungus (except for *P. infestans*, for which suspensions of 10^4^, 10^5^, and 0.3 10^6^ spores/mL were tested). For bacteria, three dilutions of the medium (3x, 30x, 300x) and three bacterial suspensions (10^6^, 10^7^, and 10^8^ bacteria/mL) were tested.

Each well was filled with one volume of culture medium, one volume of the pathogen suspension in culture medium, and one volume of water containing 2% of tween 20. The plates were then incubated in the dark at 23 °C. Pathogen growth was assessed by measuring the optic density at 630 nm with a spectrophotometer (Thermo, LabSystems Multiskan RC 351, Chantilly, VA, USA) every 24 h for 144 h. Sixteen replicates (wells) were made for each growing condition (medium and pathogen concentrations). Conditions giving the best pathogen growth are listed in Table 3, and will be the growth conditions selected to go further in the EO screening tests.

#### 2.4.2. Screening

The in vitro screening method in liquid medium is similar to the method used to determine pathogen kinetic growth (see Section 2.4.1). In 96-well ELISA plates—each well was filled with one volume of the selected medium at the optimal concentration (see Table 3), one volume of the pathogen at the optimal suspension (see Table 3), and one volume of the selected EO emulsion (see Section 2.3) at 500 and 1000 ppm (final concentration), except for *P. infestans*, for which EOs have only be tested at 1000 ppm. The plates were incubated in the dark at 23 °C. Growth was assessed by measuring the optic density at 630 nm with a spectrophotometer (Thermo, LabSystems Multiskan RC 351, Chantilly, VA, USA) after 24, 72, and 120 h (for fungi) or every 2 h during 12 h (for bacteria).

**Figure 1:**
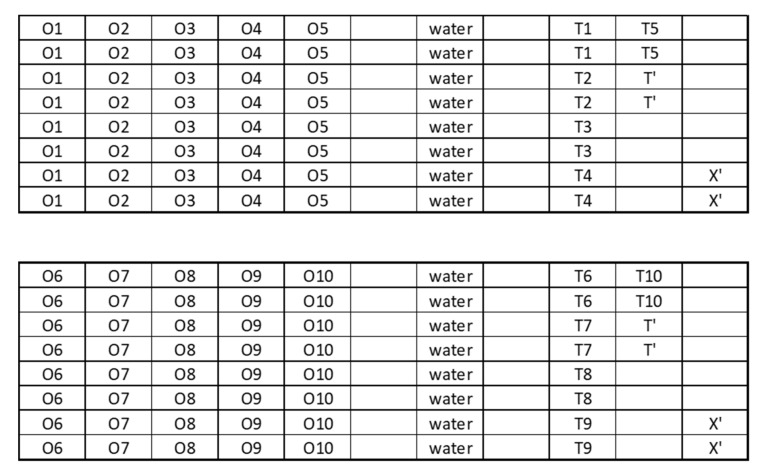
Objects repartition on the ELISA plate for an optimal screening procedure. O1 to O10 represent the tested objects (essential oils (Eos)), T1 to T10 represent negative controls (without pathogen), T’ is the culture medium only and X’ is the growth control (medium and pathogen). Eight replicates (wells) were made by object.

Figure 1 shows the wells repartition on the plate for an optimal screening procedure, minimizing the contaminations, following [16].

The efficacy of each EO against each pathogen was calculated using the following formula (1):

(1) $$\mathrm{Efficacy}\;\mathrm{of}\;\mathrm{treatment}\;n\;(\%)\;=\;\frac{\overset{´}{\left(X^{\prime }-X_{0}\right)}-\overset{´}{\left(X_{n}-X_{n0}\right)}}{\overset{´}{\left(X^{\prime }-X_{0}\right)}}\;\times \;100$$

where X’ is the optical density of the non-treated growth control at time “t”, X0 is the optical density of the non-treated growth control at time “0”, Xn is the optical density of treatment “n” at time “t” and Xn0 is the optical density of treatment “n” at time “0”. The values of the negative control Tn (negative control for treatment n: EO and medium only) and T’ (medium only) are also checked to be sure that no contaminations occurred. Heat maps were created using the “ggplot2” package of the R software using the mean of the eight replicates for each couple “EO x Pathogen x Time”.

## 3. Results

### 3.1. Evaluation of the Effect of the 90 EOs on the 10 Pathogens

#### 3.1.1. *P. expansum*

**Figure 2:**
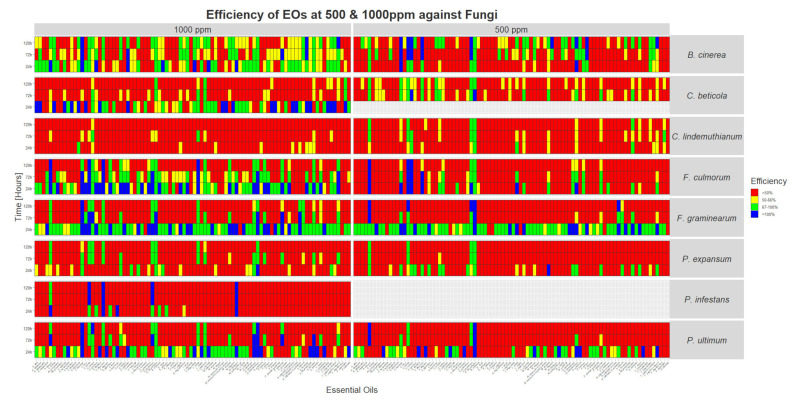
Heat map showing the efficiency of the 90 EOs at 500 and 1000 ppm on the growth of eight plant fungal pathogens after 24 to 120 h of contact in liquid medium in vitro. Red squares represent efficiencies below 50% of growth reduction. Yellow squares represent reduction of growth comprised between 50 and 66%. Efficiencies between 67 and 99% are represented by green squares, while blue squares show a complete inhibition of the organism.

At 500 ppm, 20 compounds have shown an interesting effect on *P. expansum* growth (efficacy comprised between 67 and 100%) lasting at least 24 h (see Figure 2). In general, the efficacy of the EOs at 500 ppm do not last very long (around 24 h), with some exceptions, for which the activity lasts more than 120 h: *A. sativum*, *C. cassia*, *C. zeylanicum*, and *E. caryophyllus*.

At 1000 ppm, 20 compounds have shown an efficacy comprised between 67 and 100% lasting at least 24 h. In this case, there is also an increasing number of compounds keeping high efficiencies upon time: *A. sativum*, *C. cassia*, *C. zeylanicum*, *C. citratus*, *C. flexuosus*, *Leptospermum petersonii*, *L. citrata*, *C. capitatus*, *Origanum heracleoticum*, *Origanum compactum*, and *E. caryophyllus*.

In particular, EOs of *Monarda fistulosa* at 500 ppm and *O. heracleoticum* at 1000 ppm completely inhibited *P. expansum* during the first 24 h.

#### 3.1.2. *B. cinerea*

At 500 ppm, 35 EOs have shown high activities (efficacies comprised between 67 and 100%) against *B. cinerea*, lasting at least 24 h. However, the growth inhibition was observed with a delay of at least 48 h for most of them (23/35). Moreover, EOs of *C. cassia*, *C. zeylanicum*, *C. citratus*, *C. flexuosus*, and *Pimpinella anisum* completely inhibited the pathogen growth from 72 h of contact, while EOs of *Myristica fragrans* and *Thymus vulgaris* ct. thymol showed 100% efficacies from 120 h of contact with the oil.

At 1000 ppm, the majority of the tested EOs (54) have shown efficacies between 67 and 100%. Among these, 34 showed efficiencies higher than 67%, lasting at least 72 h. EOs of *A. sativum*, *Cuminum cyminum*, *Eucalyptus dives*, *Lavendula angustifolia*, *Lavendula x burnetii*, and *Mentha pulegium* completely inhibited the growth of the pathogen the first 24 h and EO of *Copaifera officinalis* showed 100% of efficacy the first 72 h. In addition, oil from *Satureja hortensis* and *T. vulgaris* ct. thymol showed 100% efficacies from, respectively, 72 h and 120 h of contact with the EOs.

The pathogen was completely inhibited by EO of *C. capitatus* at 500 as well as 1000 ppm.

#### 3.1.3. *C. beticola*

At 500 ppm, 14 EOs have shown efficacies between 67 and 100% against *C. beticola*, lasting at least 72 h. In particular, EOs of *A. sativum*, *C. cassia*, *C. zeylanicum*, *Canarium luzonicum*, *C. capitatus*, *C. flexuosus*, and *E. Caryophyllus* have shown activities lasting more than 120 h.

At 1000 ppm, 22 EOs have been highly efficient in reducing the pathogen growth (100% inhibition during the first 24 h). Moreover, 26 more have shown inhibition between 67 and 100%, lasting at least 24 h. However, only three EOs kept a high efficacy during the whole period of screening: *L. petersonii*, *Vetiveria zizanioides*, *E. caryophyllus*.

This is also the only pathogen for which EOs of *C. cassia* and *C. zeylanicum* have efficacies lower than 50% at 1000 ppm.

#### 3.1.4. *F. culmorum*

At 500 ppm, 20 EOs showed maximal activities (67–100%) against the pathogen, lasting at least 24 h. In particular, EOs of *A. sativum*, *C. cassia*, *C. zeylanicum, C. citratus*, and *E. caryophyllus* completely inhibited the growth of *F. culmorum* up to 120 h of culture. EOs of *A. sativum*, *C. cassia*, and *C. citratus* completely inhibited the growth of *F. culmorum* for 120 h at this concentration, while EOs of *C. capitatus*, *C. zeylanicum*, *L. citrata*, and *O. heracleoticum* inhibited it completely during the first 24 h, and oil of *C. flexuosus* during the first 72 h.

At 1000 ppm, 61 EOs had efficacies comprised between 67 and 100% lasting at least 24 h. For 18 of these EOs the effect lasted for at least 120 h. Moreover, EOs of *A. sativum*, *C. cassia*, *C. flexuosus*, and *L. citrata* completely inhibited the growth of the pathogen during the 120 h of the test. In addition, 26 other EOs showed efficacies of 100% lasting at least 24 h.

#### 3.1.5. *F. graminearum*

At 500 ppm, 75 of the 90 EOs tested had efficacies comprised between 67 and 100%, lasting at least 24 h. In addition, 21 EOs provided 100% of inhibition lasting at least 24 h, including *A. sativum*, *C. cassia*, and *C. zeylanicum*.

At 1000 ppm, almost all of the EOs (78) showed efficacies superior to 67%, lasting at least 24 h. Moreover, 29 EOs provided a complete inhibition of the pathogen, lasting at least 24 h, including EOs of *C. cassia* and *C. capitatus*.

Interestingly, EOs of *E. caryophyllus* at 500 ppm and of *C. capitatus*, at 1000 ppm, completely inhibited the growth of the pathogen during 120 h.

Some EOs (*C. sinensis* and *V. fragrans* auct., among others) have shown high activities (more than 67) during the first 24 h at 500 ppm, while their maximal efficacy at 1000 ppm never exceeded 50%.

#### 3.1.6. *P. ultimum*

At 500 ppm, 37 EOs have shown efficacies between 67 and 100%, lasting at least 24 h. Interestingly, it can observed that EOs of *A. sativum* and *E. caryophyllus* completely inhibit the pathogen for at least 120 h.

At 1000 ppm, 61 EOs have efficacies greater than 67%, lasting at least 24 h, among which 12 have an activity lasting 120 h. EOs of *C. capitatus*, *C. citratus*, and *O. heracleoticum* completely inhibited the pathogen growth for at least 120 h.

#### 3.1.7. *C. lindemuthianum*

At 500, only eight EOs have shown activities greater than 67%, lasting at least 24 h. EOs of *A. sativum*, *C. cassia*, *C. zeylanicum*, and *E. caryophyllus* showed the best results over time.

At 1000 ppm, three EOs have shown activities greater than 67%, lasting at least 24 h. EOs of *A. sativum*, *C. citratus*, and *L. citrata* are the most efficient EOs in this case.

None of the oils tested provided a total inhibition of the pathogen.

#### 3.1.8. *P. infestans*

At 1000 ppm, 10 EOs showed efficacies higher than 67% lasting at least 24 h. Among these, only five EOs showed efficacies greater than 67% during 120 h. EOs of *C. cassia*, *C. flexuosus*, *C. zeylanicum*, and *M. pulegium* completely inhibited the pathogen for at least 120 h.

#### 3.1.9. *P. carotovorum* (PCC)

**Figure 3:**
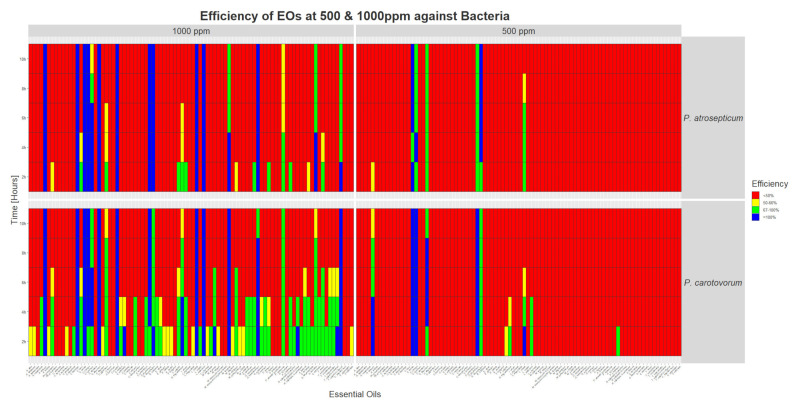
Heat map showing the efficiency of the 90 EOs at 500 and 1000 ppm on the growth of two plant bacterial pathogens after 2 to 12 h of contact in liquid medium in vitro. Red squares represent efficiencies below 50% of growth reduction. Yellow squares represent reduction of growth comprised between 50 and 66%. Efficiencies between 67 and 99% are represented by green squares, while blue squares show a complete inhibition of the organism.

At 500 ppm, four EOs are causing 100% inhibition, lasting at least 12 h: *A. sativum*, *C. capitatus*, *C. cassia*, and *C. citratus* (See Figure 3).

At 1000 ppm, the same four EOs caused a complete inhibition of the pathogen, in addition to the one of *O. heracleoticum*.

#### 3.1.10. *P. atrosepticum* (PCA)

Two EOs completely inhibited the bacterium at the two concentrations tested: *C. cassia* and *E. caryophyllus*.

At 1000 ppm, nine additional EOs caused a total inhibition: A. sativum, C. capitatus, C. citratus, C. flexuosus, Cymbopogon martini, C. zeylanicum, L. citrata, L. petersonii, and O. heracleoticum.

## 4. Discussion

In this study, the efficacy of 90 commercial essential oils against 10 plant pathogens of agronomical importance was studied.

Similar to the majority of the papers about antifungal and antibacterial effects of EOs [17,18], a dose dependent response was observed for almost all of the EOs tested in this study, the effects being stronger at 1000 ppm than at 500 ppm.

However, they were some exceptions. This is, for example, the case of *C. lindemuthianum* and *C. beticola*, for which most of the EOs showing activities were more effective at 500 ppm than at 1000 ppm. While this is not commonly found in the literature, there are some studies showing similar results [19,20]. Possible explanations are that diluted EOs could diffuse easier in aqueous environments, or that a higher rate of polymerization in concentrated EOs may reduce their antimicrobial activity [20,21].

In most of the cases, the comparison between screenings at 500 and 1000 ppm tend to show that the EO concentrations influence the time of their effectiveness on pathogens, with more concentrated formulations giving longer protection. This fact is certainly due to the high volatility of EOs.

Some EOs, such as the ones from *A. sativum*, *C. capitatus*, *C. cassia*, *C. zeylanicum*, *C. citratus*, *C. flexuosus*, *E. caryophyllus*, and *L. citrata*, have a generalist effect, and are active on several pathogens (between 7 and 10). These oils are rich in phenols, phenylpropanoids, organosulfur compounds, and/or aldehydes, known in the literature to have antifungal effects (thymol and carvacrol for *C. capitatus* [22]; neral and geranial for *C. citratus*, *C. flexuosus*, and *L. citrata* [23]; eugenol for *E. caryophyllus* and *C. zeylanicum* [24]; cinnamaldehyde for *C. cassia* and *C. zeylanicum* [25]; and diallyl di and tri-sulfide for *A. sativum* [26]).

Others, such as EOs from *C. sinensis*, *M. cajputii*, and *V. fragrans*, seem more specific, and are only active on one to three pathogens. These oils are rich in terpenes (limonene, myrcene, and pinenes for C. sinensis [27]; elemene, caryophyllene, terpinolene, humulene for *M. cajputii* [28]), and aldehydes (vanillin for *V. fragrans*) [29].

Some pathogens are more sensitive to the EOs tested, such as *B. cinerea* and the two *Fusarium* species. Some studies have already reported that fact [12,30].

Pathogens, such as *C. lindemuthianum* and *P. infestans*, seem less sensitive. Studies showing efficacies of EOs against *C. lindemuthianum* exist in the literature, but are indeed scarce: Khaledi and al [31] showed that EO of *Bunium persicum* was effective, while [32] showed effects for peppermint EO and winter green oil.

The moderate sensitivity of *P. infestans* to EOs was already reported in the literature [33] and could be explained by the fact that it is an oomycete, differing from fungi in cell wall composition and lifecycle, among others [34]. *P. ultimum*, another oomycete tested in our study, was affected by more EOs than *P. infestans*, but the observed effects were limited in time (lasting for only the first 24 h). All of the EOs having an effect on *P. infestans* also showed an activity on *P. ultimum*, except for *C. cyminum*.

For some pathogens, such as *F. graminearum*, *C. beticola*, and *P. ultimum*, the inhibition effect is very high the first 24 h, then it decreases or disappears. This result could indicate that these pathogens are more sensitive to EOs in the form of spores.

The opposite situation was observed with *B. cinerea*, where most of the efficient EOs only become active after at least 24 h of contact with the pathogen. This delayed efficacy could indicate that, in the case of this pathogen, the EOs are more efficient on the mycelium rather than on spores.

For bacteria, we observed that EOs are more efficient at 1000 ppm. PCC seem more sensitive. In general, after 10 h of contact, EOs showing an effect on PCA, which is less sensitive, are also acting on PCC. The most efficient EOs for bacteria are the same as the ones showing high activities for fungi (*C. cassia*, *E. caryophyllus*, *C. capitatus*, *A. sativum*, etc.). EOs rich in carvacrol, like the one of *C. capitatus*, were already found to be effective against PCC [35]. No oil showed specific activity against bacteria.

## 5. Conclusions

The number of studies available in the literature about fungicidal and fungistatic effects of essential oils, as well as their mechanism of action, is growing, and it is now commonly accepted that EOs have great potential in the development of new biopesticides [6,12].

In our study, 90 EOs were tested on eight fungal pathogens and two bacterial pathogens of agronomical importance. This is, to our knowledge, the largest in vitro screening of EOs made so far. This study allowed us to have a global vision of a large panel of EO efficacies, and to identify several interesting candidates, acting on a large range of pathogens: EOs of *A. sativum*, *C. capitatus*, *C. cassia*, *C. zeylanicum*, *C. citratus*, *C. flexuosus*, *E. caryophyllus*, and *L. citrata*. These oils could be promising candidates in the development of new biopesticides.

However, we have to be careful, as all of our tests have been made in vitro. The promising effects that we have observed need to be confirmed in vivo and, in particular, phytotoxic activities, which are often reported for Eos, will have to be studied [36]. We agree with [6], stating that more studies about the mode of action of EOs, the synergic effect among them or their components, and the identification of their more active components are required. More knowledge is also needed about the effect of these EO applications on the environment (beneficial organisms, soil microbiota, etc.), and on human health, even if the high volatility of EOs should minimize these effects.

## Figures and Tables

**Table 1:** List of essential oils tested in this study.

Num. Code	Plant Species	Num. Code	Plant Species	Num. Code	Plant Species
1	*Allium sativum*	31	*Eucalyptus citriodora Ct citronnellal*	61	*Corydothymus capitatus*
2	*Trachyspermum amni*	32	*Eucalyptus globulus*	62	*Origanum heracleoticum*
3	*Anethum graveolens*	33	*Eucalyptus dives CT. Piperitone*	63	*Origanum compactum*
4	*Illicum verum*	34	*Eucalyptus smithii*	64	*Cymbopogon martini var. motia*
5	*Pimpinella anisum*	35	*Eucalyptus radiata ssp radiata*	65	*Citrus paradisi*
6	*Melaleuca alternifolia*	36	*Foeniculum vulgare*	66	*Citrus aurantium ssp amara*
7	*Ocimum basilicum ssp basilicum*	37	*Gaultheria fragrantissima*	67	*Pinus pinaster*
8	*Ocimum sanctum*	38	*Pelargonium x asperum*	68	*Pinus pinaster térébenthine*
9	*Copaifera officinalis*	39	*Zingiber officinale*	69	*Pinus sylvestris*
10	*Pimenta racemosa*	40	*Laurus nobilis*	70	*Piper nigrum*
11	*Styrax benzoe*	41	*Lavendula angustifolia ssp angustifolia*	71	*Cinnamomum camphora ct cinéole*
12	*Citrus bergamia*	42	*Lavendula x burnatii clone grosso*	72	*Rosmarinus officinalis ct camphre*
13	*Fokienia hodginsii*	43	*Cymbopogon citratus*	73	*Rosmarinus officinalis ct cinéole*
14	*Aniba rosaeodora var. amazonica*	44	*Leptospermum petersonii*	74	*Rosmarinus officinalis ct verbenone*
15	*Melaleuca cajputii*	45	*Citrus aurantifolia*	75	*Amyris balsamifera*
16	*Cinnamomum cassia*	46	*Litsea citrata*	76	*Abies alba*
17	*Cinnamomum zeylanicum*	47	*Citrus reticulata*	77	*Abies balsamea*
18	*Carum carvi*	48	*Cinnamosma fragrans*	78	*Abies sibirica*
19	*Cedrus atlantica*	49	*Origanum majorana ct thujanol*	79	*Salvia lanvandulifolia*
20	*Cedrus deodara*	50	*Thymus mastichina*	80	*Salvia officinalis*
21	*Juniperus virgiana*	51	*Mentha x citrata*	81	*Satureja hortensis*
22	*Apium graveolens var. dulce*	52	*Mentha arvensis*	82	*Satureja montana*
23	*Cymbopogon nardus*	53	*Mentha x piperita*	83	*Thymus satureioides*
24	*Cymbopogon winterianus*	54	*Mentha pulegium*	84	*Thymus vulgaris ct 1 à linalol*
25	*Cymbopogon giganteus*	55	*Monarda fistulosa*	85	*Thymus vulgaris ct thymol*
26	*Citrus limon*	56	*Myristica fragrans*	86	*Thuya occidentalis*
27	*Coriandrum sativum*	57	*Myrtus communis ct cinéole*	87	*Vanilla fragrans Auct*
28	*Cuminum cymincum*	58	*Myrtus communis ct acétate de myrtényle*	88	*Cymbopogon flexuosus*
29	*Cupressus sempervirens var. stricta*	59	*Melaleuca quinquenervia ct cinéole*	89	*Vetiveria zizanoides*
30	*Canarium luzonicum*	60	*Citrus sinensis*	90	*Eugenia caryophyllus*

**Table 2:** List of the pathogens tested in this study and their culture conditions.

Host Plant/Environment	Pathogen	Culture Conditions (Medium, Temperature (°C))
Wheat	*Fusarium graminearum*	PDA, 23 °C
	*Fusarium culmorum*	V8, 23 °C
Sugar beet	*Cercospora beticola*	V8, 23 °C
Potato (tuber)	*Phytophthora infestans*	V8, 16 °C
	*Pectobacterium carotovorum*	LB-Agar, 23 °C
	*Pectobacterium atrosepticum*	LB-Agar, 23 °C
Apple and pear (fruit)	*Botrytis cinerea*	PDA, 23 °C
	*Penicillium expansum*	PDA, 23 °C
Bean	*Colletotrichum lindemuthianum*	V8, 23 °C
Soils	*Pythium ultimum*	PDA, 23 °C

PDA (Potato dextrose agar); LB-Agar (Luria-Bertani-agar).

**Table 3:** Pathogen growth conditions selected for the screening tests.

Pathogen	Selected Growth Conditions
*Fusarium graminearum*	3 times diluted PDB/10^5^ spores/mL
*Fusarium culmorum*	3 times diluted V8/10^5^ spores/mL
*Cercospora beticola*	3 times diluted V8/10^5^ spores/mL
*Phytophthora infestans*	300 times diluted V8/0.3 10^6^ spores/mL
*Pectobacterium carotovorum*	3 times diluted LB/10^7^ CFU/mL
*Pectobacterium atrosepticum*	3 times diluted LB/10^7^ CFU/mL
*Penicillium expansum*	3 times diluted PDB/10^5^ spores/mL
*Botrytis cinerea*	3 times diluted PDB/10^5^ spores/mL
*Colletotrichum lindemuthianum*	3 times diluted V8/10^6^ spores/mL
*Pythium ultimum*	3 times diluted PDB/10^5^ spores/mL
